# A novel framework for storage assignment optimization inspired by finite element method

**DOI:** 10.7717/peerj-cs.378

**Published:** 2021-02-16

**Authors:** Seyed-Kourosh Tabatabaei, Omid Fatahi Valilai, Ali Abedian, Mohammad Khalilzadeh

**Affiliations:** 1 Industrial Engineering Group, Faculty of Engineering, Science and Research Branch, Islamic Azad University, Tehran, Iran; 2 Mathematics & Logistics Department, Jacobs University Bremen, Bremen, Bremen, Germany; 3 Aerospace Engineering Department, Sharif University of Technology, Tehran, Tehran, Iran

**Keywords:** Warehouse, Storage assignment problem, Industry 4.0, Finite Element Method (FEM)

## Abstract

Considering necessary fundamental and structural changes in the production and manufacturing industries to fulfill the industry 4.0 paradigm, the proposal of new ideas and frameworks for operations management of production and manufacturing system is inevitable. This research focuses on traditional methods proposed for storage assignment problem and struggles for new methods and definitions for industry 4.0 based storage assignment concepts. At the first step, the paper proposes a new definition of storage assignment and layout problem for fulfilling storage mechanism agility in terms of automated store and retrieval process (AS/RS) in modern inventories. Then considering the shortcomings of traditional algorithms for storage assignment problem, the paper contributes a new algorithm called SAO/FEM (storage assignment optimization technique), inspired from mechanical engineering discipline for analysis and optimization of storage assignment problem. The proposed new algorithm about stress distribution analogy, and the help of the Finite Element Method and minimum total potential energy theory, proposes a new model for storage assignment optimization. The efficiency of the proposed algorithm in terms of calculation time and the best answer investigated through numerical examples. The article has developed an application for SAO/FEM algorithm as a value creation module and applied new optimized storage positioning in the warehouses.

## Introduction

The shift from the era of simple digitization (Third Industrial Revolution) to innovative hybrid technologies (Fourth Industrial Revolution) has forced the production and service system to look for innovative methods to enhance their traditional working process models (Delaram & Valilai, 2017; Delaram & Valilai, 2018a; Aghamohammadzadeh, Malek & Valilai, 2019). The business leaders and senior executives have realized this force and are struggling to make the changes in their business environment by challenging the assumptions of their firms working teams and working procedures and with continuous innovative changes in the processes. Manufacturing industries are now shifting from mass production to custom production (Cao et al., 2017; Zhang, Wu & Ma, 2017). The fourth revolution in industry has proposed the paradigm that requires seamless integration among all the stakeholders in the manufacturing processes while they fulfill the requirements of a dynamic behavior in process execution. This revolution emphasizes for system agility, productivity and sustainability (energy) and Waste avoidance (Ferrera et al., 2017; Müller, Kiel & Voigt, 2018).

Warehouses and distribution centers play an important role in supply chains of companies. Considering the Information Technology impact on bringing out new business models, the expectations from the warehouses have been changed for enabling more agility and dynamic (Delaram & Valilai, 2016; Delaram & Valilai, 2018b; Delaram & Valilai, 2019). In order to optimize the design and reduce operational costs, many operating warehouse companies have invested on automated operations and many academicians have conducted wide variety of research studies (Houshmand & Valilai, 2013; Valilai & Houshmand, 2014). There are different operation research models and software algorithms to take care of solving different class of problems, like slot storage allocation, pick location allocation, replenishments timing, reserve and send storage locations, and routing of an AS/RS which have been recognized as classic models in this research (Hausman, Schwarz & Graves, 1976; Roodbergen & Vis, 2009).

As a classical AS/RS storage is static, a calculation related to the optimization, focuses on cost equations and behaves mainly through reducing the factors of time and distance of movements, so the transport factors of goods try to carry out the shortest path and optimal layout of the storage elements in the lowest time (Sadiq, Landers & Don Taylor, 1996; Moon & Kim, 2001). Nowadays as quick and time-based ordering are increasing, the warehouses must adapt to support the rate of demands, ordering fulfillment and the factor of inquiry (FOI) of real market requirements. So, many changes must be accomplished in the classical models, like increasing the variety of goods, decreasing the dimensions of goods or increasing the storage space divisions, and the strategy of storage assignment. This change affects the number of variables and finally affects cost-related equations, so it will be difficult to solve the layout and storage problems easily and in a reasonable time (Brezovnik et al., 2015).

In this regard, it is necessary to consider the dynamic storage elements are no longer the same as the classical model and have variable spatial coordinates at any time (depending on speed of use, input and output frequency, etc.) due to cost effects, it is necessary to locate the elements in proper locations in the warehouse. This requires an innovative algorithm that is able to give right resolution and accuracy in spatial coordinates while calculating the ideal location rapidly.

This research first, proposes a new definition of storage assignment and layout method considering the requirements for dynamic storage elements assignment. Then it creates a new algorithm inspired from mechanical engineering fields for analysis and optimization of storage to end constrains of some of the existing algorithms. The new algorithm called SAO/FEM (Storage Assignment Optimization technique, by the help of Finite Element Method and total potential energy theory), proposes a new model for storage assignment optimization. The paper has developed an application for SAO/FEM algorithm and applied the new optimization storage positioning to some warehouses and conducted comparisons with the classical method, first to prove the feasibility and performance of the new algorithm then to present the new advantages. In other words, this algorithm acts as a value-added module to meet the requirements of the fourth generation of the industry in the field of layout and warehousing. As such, this model can satisfy the dynamic storage situation and can respond to the rapid behavior resulting from high-speed production or service systems.

## Literature Review

AS/RS design requirements such as strategic location and choosing the right space for warehouse construction are considered with the rigidity of the hardware system for warehouse operations management to fulfill the optimal warehouse model (Rosenblatt, Roll & Vered Zyser, 1993). Selecting the type of warehouse and its operational variables, warehouse configuration, software and hardware systems are among the most important factors shaping the performance of the warehouse. Obviously, the optimal performance of a warehouse always depends on the strategy for handling the material flow inside/outside for optimal performance; so that the warehouse will not fail in future in terms of responsiveness for material store and retrieval operations (Gagliardi, Renaud & Ruiz, 2012).

Literature review on the classical models for AS/RS demonstrates that many research studies have been proposed to optimize warehouse operation through exact or numerical methods. Multivariate problems are solved by approximate, heuristic or Meta heuristic methods. Different research fields on the classic warehouse model and its dependencies such as layout problem is almost saturated, and many of the old definitions need to be redefined based on the new requirements of agile store and retrieval processes (Rosenblatt, Roll & Vered Zyser, 1993; Roodbergen & Vis, 2009; Gagliardi, Renaud & Ruiz, 2012; Gagliardi, Renaud & Ruiz, 2014; Cao & Zhang, 2017; Hamzaoui et al., 2019; Huh et al., 2019; Reyes, Solano-Charris & Montoya-Torres, 2019). Moreover, in the area of warehouse physical design and resource configuration and storage strategies in warehouse design, most of the research studies have focused on calculating Single Storage Rack layout and on storage capacity or storage position. There has been a considerable gap for optimal position of the entrance and exit doors (Rosenblatt, Roll & Vered Zyser, 1993). Also, in the field of warehouse operations control, most of the researchers have involved one or a few control mechanisms for order processing in the design of their proposed warehouse structure (Hamzaoui et al., 2019). Although the structural design and control system of warehouse are highly interdependent, in many research studies both have been examined individually and independently. It demonstrates the challenge of mutual optimal number of the goods and the location of the I/O points determination (Hausman, Schwarz & Graves, 1976; Bessenouci, Sari & Ghomri, 2012; Gagliardi, Renaud & Ruiz, 2012; Gagliardi, Renaud & Ruiz, 2014; Hamzaoui & Sari, 2015; Guo, Yu & De Koster, 2016; Hamzaoui et al., 2019).

### Storage assignment literature

The literature suggests various methods for products storage location assignment. There are five basic storage assignment policies for AS/RSs (Roodbergen & Vis, 2009; Faber, De Koster & Smidts, 2013; Ekren et al., 2014). These rules are:

• Dedicated storage assignment
• Random storage assignment
• Open storage assignment in closest location
• Full-turnover-based storage assignment
• Class-based storage assignment

In dedicated storage method, for each product category a fixed location is considered. And at this location replenishments of that product are considered. The main disadvantage will be space requirements and low space utilization. This is due to limitation of this method in considering the out of stock products in the model and reserve locations for them. Also, to be able to fulfill the placement for probable maximum inventory level, the model should reserve enough spaces based on each product type (Heskett, 1963; Heskett, 1964; Zhang, Wu & Ma, 2017).

For random storage in all empty locations, equal probability exists for an incoming load assigned to it. The product is assigned to first free location which is encountered. The product demand frequency is considered for full-turnover storage policy for assigning storage locations near to the I/O-points. So, the easiest accessible locations are considered for high frequently demanding products. In addition, the products called slow-moving are located farther away from the I/O-point. For this rule, the product turnover frequencies will an important assumption which should be known first (Roodbergen & Vis, 2009).

The cube-per-order index (COI) rule presented by Heskett (Eynan & Rosenblatt, 1993; Moon & Kim, 2001; Roodbergen & Vis, 2009). Explains the full-turnover storage method. The COI is defined by the ratio of needed storage space for products to amount of product requests for a load in a specific period. The COI rule manages the assignment policy by placing loads with the lowest COI to the nearest locations to the I/O-point. The main challenge is that the demand frequencies and the product assortment are usually changing. The method will encounter challenge when the frequency of demands for products are changing dynamically or a new product is added to the system. It requires a huge number of calculations for reassignment of products and fulfilling the full turnover rule.

To reduce periodic repositioning and also fulfilling space requirements, class-based storage can be considered. The literature studies show that class-based storage method is the most consistent policy for storage assignment (Hausman, Schwarz & Graves, 1976; Eynan & Rosenblatt, 1994). Class based storage method splits the available space in the warehouse into several sectors. Then the demand frequency is considered for each item and items are subsequently assigned to the sectors. Inside a sector area, the assignment is completed randomly. So, the full turnover storage policy is a class-based policy which only consist one item per class. There is a so-called ABC storage which is a class-based storage with three classes (Graves, Hausman & Schwarz, 1977). The A-items are with the highest turnover and then in descending order of turnover frequency products are called B-items, and so on. The main merit of class-based storage is its efficiency for assigning high frequency products close to I/O-points and also benefiting from the flexibility of random storage method and being applicable for low storage space conditions.

There are three decision variables for fulfilling the class-based storage in an AS/RS:

• Zone division (i.e., in which the number of classes are determined),
• Zone sizing (i.e., the number of products, assigning to each zone),
• Zone positioning (i.e., defining the location each of the zones).

To include storage boundaries for above mentioned classes, several procedures have been found in the literature for zone sizing. According to the Eynan and Rosenblatt’s research studies (Guenov & Raeside, 1992; Eynan & Rosenblatt, 1994), a relatively small number of classes, less than 10, gain most of the probable savings in travel times as compared to full-turnover storage. However, there are limited number of classes to three in real case problems.

For zone positioning several strategies exist. Research studies (Graves, Hausman & Schwarz, 1977) have proven that when single command scheduling is considered in square-in-time frames, the optimal solution for classes A, B and C with square-in-time boundaries will be an L-shaped configuration. Also, in other research studies (Kulturel et al., 1999) it has been demonstrated by simulation there will be optimality for L-shaped configuration for dual command scheduling, in square-in-time frames. Another research (Aldenderfer & Blashfield, 1984) also studied dual command scheduling and compared three different zone shapes. Their conclusion is that the location of the I/O-point affects the performance of proposed shapes while none will be superior to the others.

Another research study (Kaylan & Medeiros, 1988) has discussed the application of single command scheduling in “n” classes in rectangular frames and combined “n-2” L-shaped zones in layout, i.e., a rectangular zone for class “n” and a transient region for class “n-1”.

### Performance of storage assignment rules

An analytical approach for checking control rules can be travel time estimation in scheduling for different command types in different types of AS/RS configurations (Van Den Berg & Gademann, 2000). Using the simulation approach, the stochastic conditions were focused and more comprehensive studies have been accomplished (Goetschalckx & Ratliff, 1990; Jaikumar & Solomon, 1990; Muralidharan, Linn & Pandit, 1995; Malmborg, 1996; Malmborg, 1998; Kulturel et al., 1999). Before simulation study, a trade-off storage policy was developed for small systems and then based on that for unit load the rough comparisons of different policies were accomplished. Results from both simulation and analytical studies stated that class-based storage assignment and the full-turnover based outperform random storage policy. In Moon & Kim (2001), considering duration of stay policy, a comparison was made by a three class-based policy which was originally introduced informer studies for shared storage policies in Sadiq, Landers & Don Taylor (1996). Storage locations are assigned based on a policy which consider the nearest locations to the I/O-point for short staying products in the storage space while duration of stay policy was considered. In cases where the number of the product variety is fewer, the three-class-based policy overtakes the stay policy duration.

For today’s dynamic environment (Roodbergen & Vis, 2009), there is an essential need for proposing storage assignment policies in AS/RS system. This fulfills the requirements for having the required performance level for dynamic storage management (Heragu et al., 2005). The COI policy can be considered as an acceptable policy assignment when the logistics and handling frequency parameters for demand of products are static. (Moon and Kim) (Heragu, 2008) in a simulation study showed that when the production quantity of each storage items is fluctuating by time, there will requirements for item re-location. Sadiq (Roodbergen & Vis, 2006) proposed a dynamic storage assignment policy for reassignments of products in storage locations for short lifecycle products and also the mixtures which were dynamically changing. The research applied a prediction model for future product mixtures by using correlated demand of products as well as product demand. Then a dynamic policy for storage assignment was focused targeting to decrease the order processing times (relocating times and order picking times). The results showed that the proposed dynamic policy fulfilled the static COI shortcomings for dynamic conditions.

Considering the related research studies, by using simulation and analytical methods different storage assignment policies have been compared. Most research studies are limited by considering one-way passage AS/RSs which include only one I/O-point. There is considerable gap for storage assignment policies for other kinds of multiple I/O-points configurations or multiple shuttles equipped AS/RSs. By the increase of variables like number of I/O-points or number of classes in the warehouse, the complexity of forming optimal cost equation and solve parameters increases drastically (Barkanov, 2001). Besides the demands on storage assignment are changing rather than the level of automation, cost effectiveness and maximum throughput, other characteristics that are more important today are flexibility, configurability and high availability. Due to virtual development, the time needed to introduce a new model in the market has been shortened rapidly and the diversity of products is dramatically increasing, also shortening the product life cycle, it shortened planning horizons and smaller amortization periods result in smaller investments. Also, customer request for more individual products leads to mass customization, which in return it leads to smaller quantities of the product in almost every sector of the consumer market. To cope with the fast-changing demands, classic models and related algorithms are not effective and competent and so a new model has to be developed which needs to be dynamic, flexible and alterable.

### Storage assignment models

As it was previously said, generally there are five major policies for storage assignment in an AS/RS. The most comprehensive and practical applications currently being used in warehouses are Dedicated storage assignment, Full-turnover-based storage assignment, Class-based storage assignment, or Integrated Optimal use of these policies.

In the first case, any commodity type always belongs to a particular location, but its main drawback is the necessity for large space for storage. Thus, little space left for storage equipment which is generally not mechanized. However, storage of large or heavy- volume goods is the most important advantage of this case.

In the second case, locating construction is based on the frequency of demand for goods, so the goods that are most needed are placed in the places close to entry/exit doors that provide better, simpler and faster access to those goods. In this method, as was explained before COI index rule for each entry is applied. This rule places lower loads with the less COI index in the nearest locations to entry or exit doors. The drawback here refers to any change in demand frequency or product definition (new load) which causes a large volume of displacements and possible layout changes in the warehouse.

In the third case, the space available in the warehouse is divided into several parts and the loads subsequently classified into each space based on demand frequency (each space is allocated only to a specific class), and the location of each class inside, can be randomly assigned. In this way in order to better managing space and time, mainly three classes of goods were considered (ABC Storage) that ranging from Class A (consuming) to Class C (low consumption). The advantage of this case is warehouse flexibility due to random storage of goods (in each class) and optimal use of space in warehouses. The cost model, developed by incorporating the benefits of these policies and currently being used in the literature and layout calculations, is set to minimize the cost of moving items to entry and exit points. The cost per pair of goods/storage space is calculated as the product of three factors (Ventsel & Krauthammer, 2001).

• Frequency of trips to each I/O point.
• The distance being traversed to any I/O point.
• Cost per unit distance (where cost per unit distance is different for different combinations of entry points and exit points for different products).

### Dedicated storage policy model

In this model (which is called first model), a warehouse has “p” I/O points through which “m” items enter and leave the warehouse. The items are stored in one of “n” storage spaces or locations. Each location requires the same storage space, and it is known that item i requires s_i_ storage spaces so ideally

(1) \begin{eqnarray*}\sum _{i=1}^{m}{s}_{i=n}\end{eqnarray*}

There are *f*_*ik*_ trips of item *i* through I/O point *k*, the cost of moving a unit load of item *i* a unit distance through I/O point *k* is *c*_*ik*_ and the distance of storage space *j* from I/O point *k* is *d*_*kj*_, given a binary decision variable *x*_*ij*_ specifies whether or not item *i* is assigned to the storage space *j*, so to formulate a model to assign the items to storage spaces in the way that minimize the cost of moving the items in and out of the I/O points one would have:

(2) \begin{eqnarray*}\text{minimize} \sum _{i=1}^{m}\sum _{j=1}^{n} \left( \sum _{k=1}^{p}{c}_{ik}{f}_{ik}{d}_{kj}/{s}_{i} \right) \mathrm{}{x}_{ij}\end{eqnarray*}

Subjected to:

(3) \begin{eqnarray*}\sum _{j=1}^{n}{x}_{ij}={s}_{i}i=1,2,\ldots ,m\end{eqnarray*}

(4) \begin{eqnarray*}\sum _{i=1}^{m}{x}_{ij}=1 j=1,2,\ldots ,n\end{eqnarray*}

(5) \begin{eqnarray*}{x}_{ij}=0~or~1~i=1,2,\ldots ,m;j=1,2,\ldots ,n.\end{eqnarray*}

Substituting ${w}_{ij}={\mathop{\sum }\nolimits }_{k=1}^{p}{c}_{ik}{f}_{ik}{d}_{kj}/{s}_{i}$

The objective function will be

(6) \begin{eqnarray*}Minimize\sum _{i=1}^{m}\sum _{j=1}^{n}{w}_{ij}{x}_{ij}\end{eqnarray*}

### COI policy model under certain conditions (Zhang & Kratzig, 1995)

In this model (second model), a special case of the design model for dedicated storage policy is considered.in this case all the items use the I/O point in the same proportion and the cost of moving a unit load of item *i* is independent of the I/O point. *P*_*k*_ isdefined as the percentage of trips through I/O point “k” where *k* = 1, 2, …, *p* (for any item because all the items use the I/O points in the same proportion). Due to the additional constraints included in this model, there is no need for the first subscript in *f*_*ik*_ or in *C*_*ik*_. Therefor *f*_*ik*_, *C*_*ik*_ can be replaced by *f*_*i*_, *C*_*i*_ respectively, and the model may be formulated as follows:

(7) \begin{eqnarray*}\text{minimize} \sum _{i=1}^{m}\sum _{j=1}^{n} \left( \sum _{k=1}^{p}{c}_{i}{f}_{i}{P}_{k}{d}_{j}/{s}_{i} \right) \mathrm{}{x}_{ij}\end{eqnarray*}

Subjected to constraints Eqs. (3)–(5).

Substituting

\begin{eqnarray*}{w}_{j}=\sum _{K=1}^{P}{P}_{k}{d}_{j} \end{eqnarray*}

The objective function can write as:

(8) \begin{eqnarray*}Minimize\sum _{j=1}^{n}\sum _{j=1}^{n} \frac{{c}_{i}{f}_{i}}{{s}_{i}} {w}_{j}{x}_{ij}\end{eqnarray*}

The model given by expression Eq. (8) and constraints Eqs. (3)–(5), is easier to be solved in comparison with the first model given in expression Eq. (6) and does not require the use of transportation algorithm. It involves rearranging the “cost” term $ \frac{{T}_{i}}{{s}_{i}} $ (if *c*_*i*_*f*_*i*_ = *Ti*) for each item i (*i* = 1,2,…,m). Also the “distance” term *w*_*j*_ for each storage space *j*(*j* = 1, 2, …, *n*) in non-increasing and non-decreasing order, respectively, and matching the item *i* corresponding to the first element in the ordered “cost” list with the storage space corresponding to the first *S*_*i*_, elements in the ordered “distance” list, the second item, *j* in the “cost” list with the storage space corresponding to the next *S*_*i*_, elements in the ordered “distance” list and so on until all the items are assigned to all the storage spaces. This is exactly what the COI policy except it calculates the inverse of the “cost” term, COI, that is, the storage space requirement divided by the cost incurred as a result of handling item *i* (or the frequency of item *i*)*,* and orders the elements in non-decreasing order of their COI values . Thereby producing the same result as the preceding algorithm.

**Figure 1:**
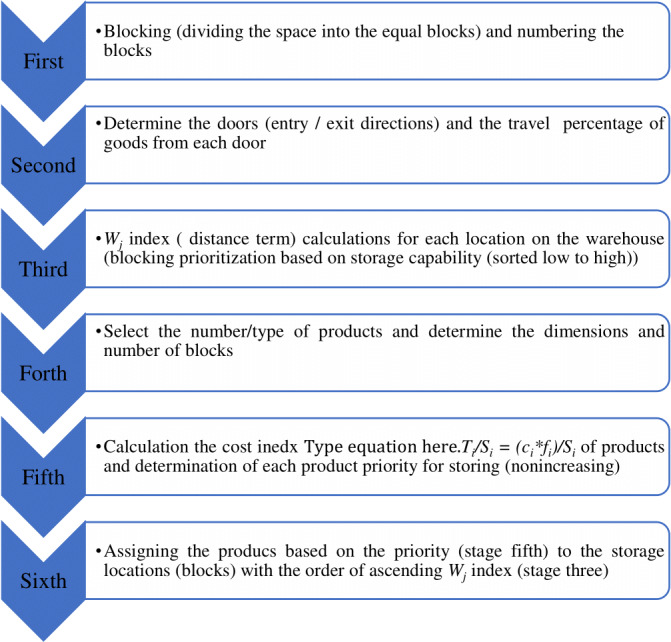
Classical algorithm for storage assignment base on COI model index.

As defined in the algorithm (Fig. 1), the objective function has two parts and the storage optimization on the layout is performed in two stages: The first stage involves partitioning of the storage space, the calculation of distance index (*W*_*j*_) will be done on the storage area and the contribution of each unit in the storage division will be clear, the best location is the one that has the lowest index (*Wj*). It is evident that the index value will change according to the location of each block at the warehouse, the number of divisions in the warehouse level, the number of doors, the distance of the doors from the divisions, and the trip percentage of each good (Persson & Strang, 2004).

The second stage, is the calculations of (*Ti/Si*) index which is called cost index, any good that has higher cost per unit has higher priority for storage on nearest location. The computation has to be done in a way that the total cost of moving items is minimum and items with higher consumption factor are placed at optimum storage positions.

## Storage Assignment Example

The following problem is presented the second model:

### Example 1

A warehouse with 16,000 units area is available as follows: The area size 16,000 units, has two I/O points with 30% and

**Figure 2:**
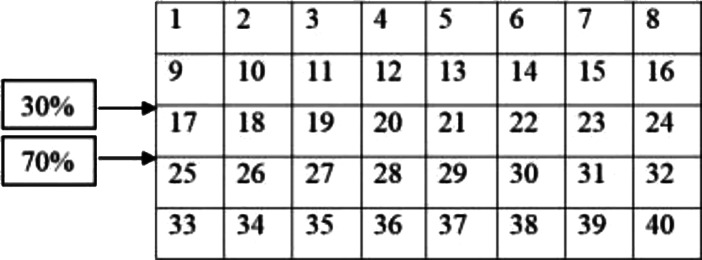
Warehouse area with 40 divisions.

70% input and exit logs on one side and with 40 equal divisions on the area (20 × 20 = 400) (Fig. 2).

**Figure 3:**
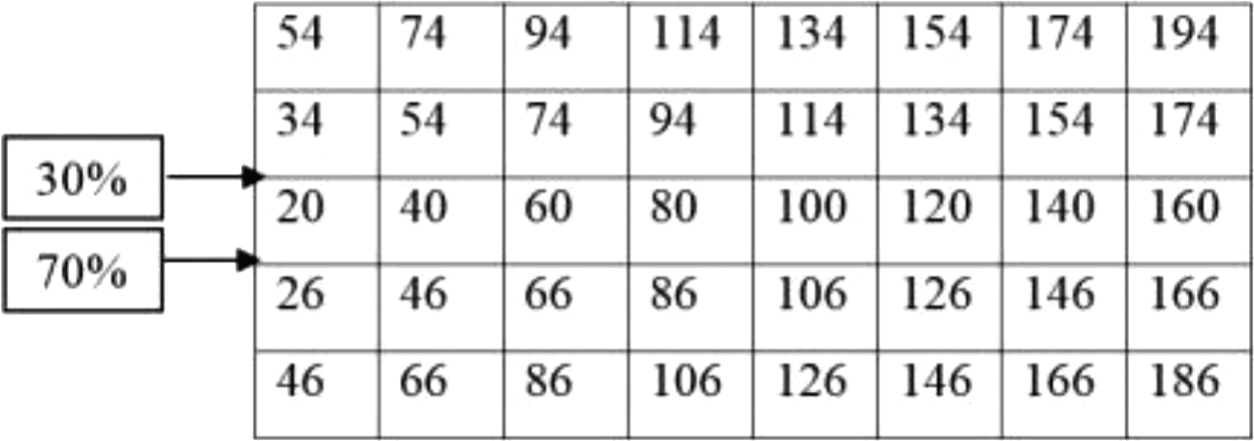
Implementation of the optimal distance index to each location (unit) of the warehouse.

Three products of A and B and C are to be stored base on Table 1 specifications:

After calculating the first step of the classical storage algorithm, and implementation of the distance index on the warehouse area, the results are presented in Fig. 3.

**Figure 4:**
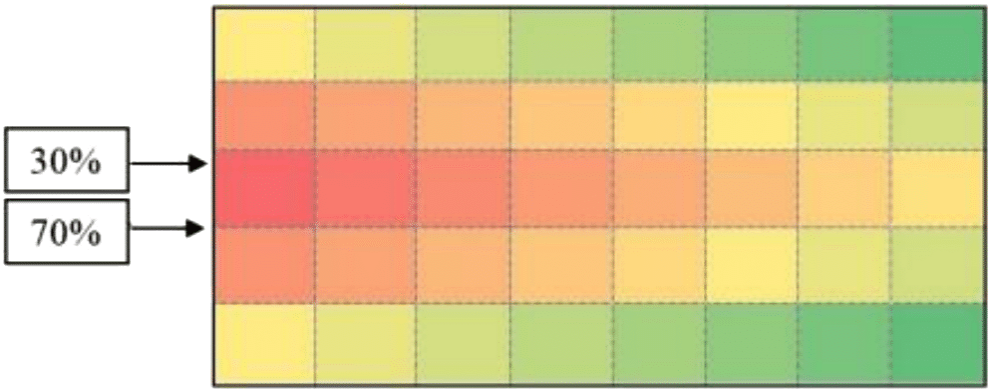
Storage assignment priority distance spectrum on warehouse area of Fig. 3.

**Figure 5:**
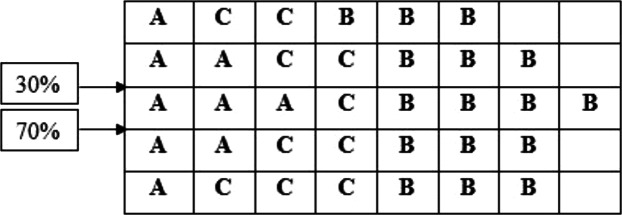
Placement of products according to the priority order (A > C > B).

**Figure 6:**
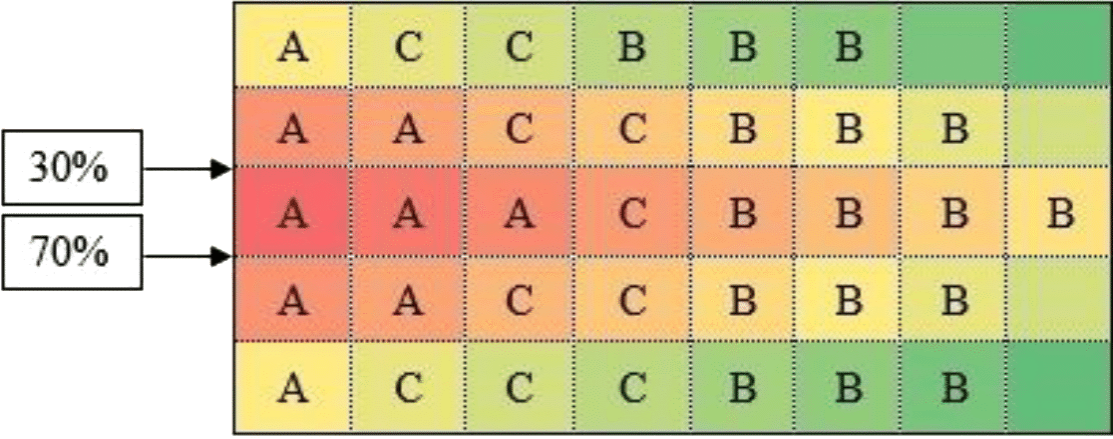
Placement of products with the products’. priority order (A > C > B) on optimal distance spectrum of storage priority.

According to Fig. 3, calculations of the spectrum of Fig. 4 shows the prior assignment locations are near to I/O point and as far as getting out of the I/O points, the storage assignment priority is reduced. The algorithm is then executed based on the computation of the Table 1 and outputs are placed in priority order on the partitions by the non–descending trend on the warehouse (Figs. 4–6).

## Methodology

### Finithe element method (FEM)

Finite element method is a numerical method for solving the differential equations governing the real problem presented in the form of a mathematical model. To achieve accurate, strain and stress result of a structure, the equilibrium equations must be established, and in addition the boundary conditions of the problem have to be satisfied (NguyenVan, Mai-Duy & Tran-Cong , 2007) .

**Table 1:** The assumptions about the specifications of the storage and the calculation of commodity storage priorities.

Products	Ti (Frequency)	Ai (Area)	Si (Block numbers)	Ti/Si	Storage priority
A	750	3,600	9	83.33	1
B	900	6,400	16	56.25	3
C	800	4,000	10	80	2

**Figure 7:**
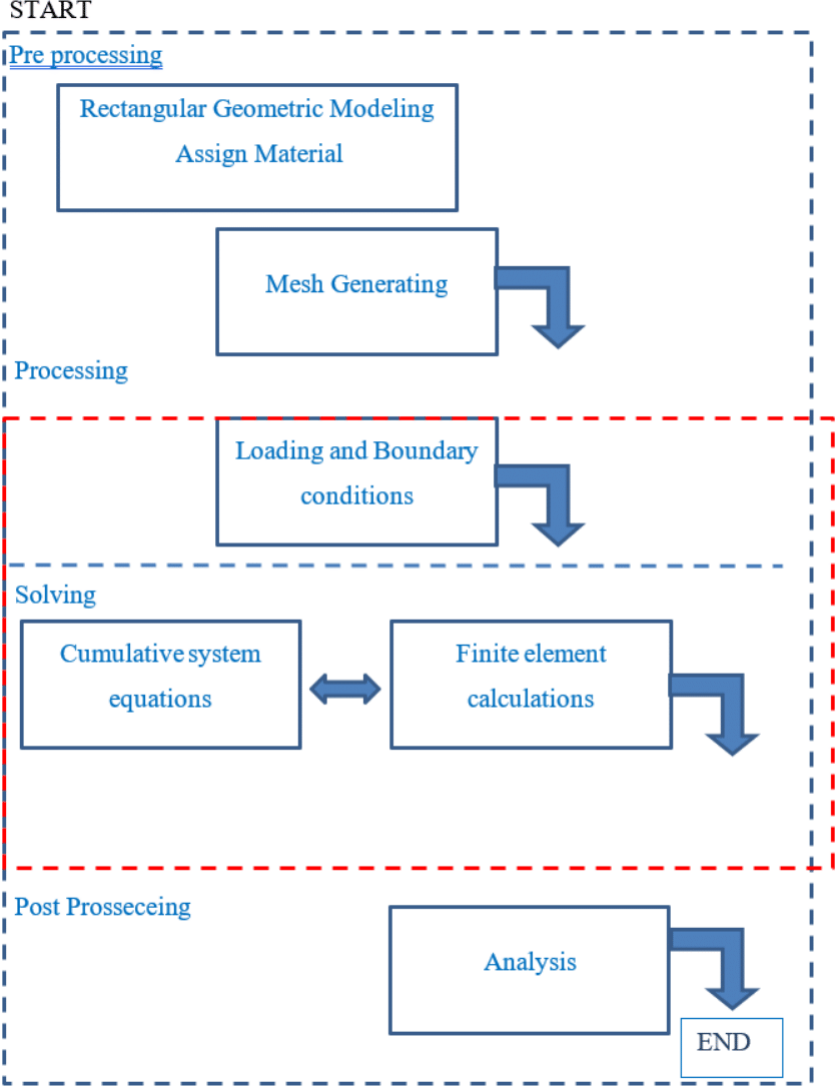
Schematic diagram of the Finite element implementation.

Solving equilibrium equations along with the establishment of boundary conditions and adaptation in non-complex problems is not hard work. However, if it is faced with a complex problem (from the point of view of geometry, material behavior, loading, etc.), the exact solution of the above equations is practically very difficult and, in some ways, it is almost impossible (Fig. 7) (Nadal et al., 2013).

Therefore, the finite element method is an accepted and efficient method for solving differential equations governing the behavior of the object (problem), whose efficiency and speed of operation have been practically proved and generally in accordance with the following steps (Zhang & Kratzig, 1995; Persson & Strang, 2004; NguyenVan, Mai-Duy & Tran-Cong , 2007; Nadal et al., 2013).

#### General description of finite element method

1. The discretization of the Mesh structure
2. Presenting the physical behavior governing the element quantitatively.
3. Select an interpolated model or suitable transportation model
4. Extraction of hardness matrix and elemental force vectors.
5. Composition of the equations of elements for extraction of general equilibrium equations.
6. Calculating and extracting displacements in nodes.
7. Calculation of stresses and strains of elements.

#### Finite element method optimization algorithm steps

1. Preprocessing, first Geometric model of the rectangular plane and mesh model of the problem is constructed. Then the loading and boundary conditions are applied to the model.
2. Solving the finite element model, the integration of the system equations and solving the general equations is carried out at this stage.
3. Post processing, preparing and displaying results.

### Stress in thin plates

Man-made objects are often made from stock plates of various materials by operations that do not change their essentially two-dimensional character, like cutting, drilling, gentle bending and welding along the edges. The stress description in thin plates is simplified by assuming the plate with two-dimension surfaces and neglecting the three-dimensional bodies. With this assumption, particles are considered to cover the plate’s surface, so that the boundary between adjacent particles will be tiny line. The surfaces of particles are located such that their normal vectors are straight through the plate. The Stress can be defined as the result of internal forces between two adjacent particles through their overlapping line element divided by the length of that line. Usually, some elements of the stress tensor are ignored, however, since particles are only considered through two-dimension, the third dimension cannot be neglected for the torque among particles positioned in neighbor location (Chandrupatla et al., 2002; Huston & Josephs, 2008; Smith, 2013).

### Conceptual model

In this study, we try to design and develop mapping as an optimal layout allocation model based on natural stress distribution algorithm (considering the behavioral similarity of stress extension pattern), using finite element computational and analytical method. The potential energy minimum theory is used for the analysis and based on the new heuristic algorithm; the claimed capabilities of the new model are verified.

**Figure 8:**
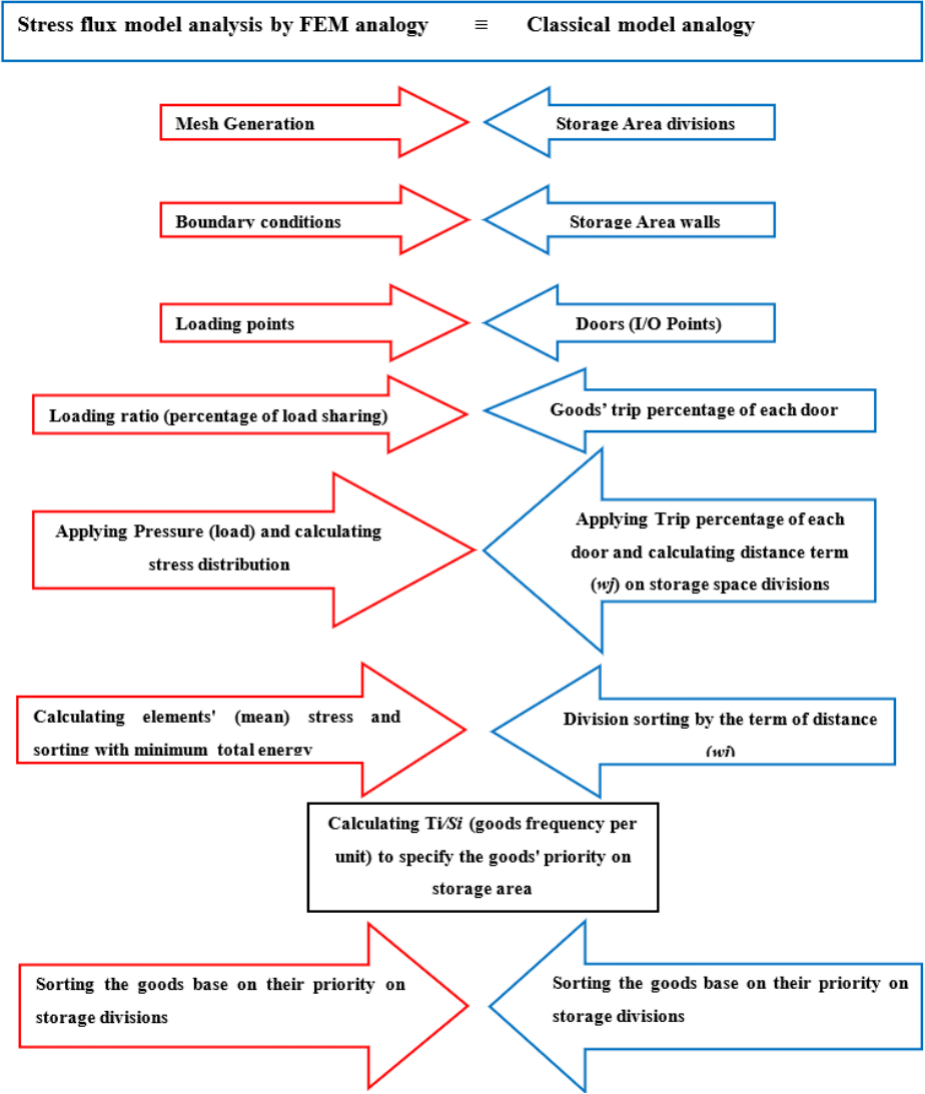
Conceptual mapping model of classical and finite element method.

To use stress distribution analogy for storage assignment, first it is needed to define the conceptual mapping model of classical and finite element method, to match up some of the definitions, rules and activities, then an algorithm needed to be designed and implemented to prove that the new model is efficient and shows its new advantages as shown in Table 2 and Fig. 8.

To elaborate this mapping as illustrated in Table 2, first the properties of the classical model are defined as follows:

A-1) the warehouse is a rectangle.

A-2) The storage area of the warehouse is unobstructed, shelving and surface level.

A-3) the storage area has four walls (parallel to each other).

A-4) the storage area is divided into smaller, uniform areas (square, in this model) according to the requirements of the warehouse.

A-5) in this model, I/O doors are defined and installed on parallel (facing) walls.

A-6) It is possible to define two doors (I/O points) solely on parallel walls, and the doors are logically defined on the wall spacing most separately on the marginal points of the warehouse surface segmentation.

A-7) the storage area is loading or unloading at one period.

A-8) in this model, according to the number of the doors, the share of goods entering / leaving each door is defined as a percentage of the total supply capacity of the warehouse.

A-9) in a coordinated manner, over a period, the warehouse (all doors) is in the state of entry or exit.

Considering the abovementioned properties, the paper presents all the classical features in FEM perspective with the least variations. So, in the new proposed model the features are defined as:

B-1) a rectangular plate is considered as the surface of the storage area.

B-2) the plate is made of soft, homogeneous metal with a low thickness.

B-3) the boundary conditions are applied to the plate in such a way as the sides with no entry or exit doors (I/O points) restricted.

B-4) the warehouse surface is meshed with square elements according to the classical model classification.

B-5) in the new model, the entry/exit doors are defined on the pair of parallel sides with the greatest distance.

B-6) loading points on the same positions of classical I/O positions are defined on the nodes resulting from the meshing on non-restricted sides.

B-7) loading at all points over a period is merely compressive or tensile.

B-8) in the new model, the share of loading on I/O points are calculated as a percentage of unit load, like the proportion of entering/leaving of each door in the classical model.

**Table 2:** Comparison of the paper idea with classical model in the literature.

The FMEA domain		The classical model in the literature
Stress distribution & Finite Element Scope	≡	Classic Model Scope
Plate surface meshing	≡	Dividing storage space
Boundary conditions	≡	Warehouse walls
Loading points	≡	Warehouse I/O points
Point Load Ratio (per unit load)	≡	each door’s entry/exit percentage of goods
stress calculation in nodes and elements’ arrangement based on minimum potential energy from loading points	≡	Implementation of index Wj
Calculation Ti/Si (Determination of each goods’ priority of storage)		
Sorting the goods by storage priority		

### simulation and analysis

**Figure 9:**
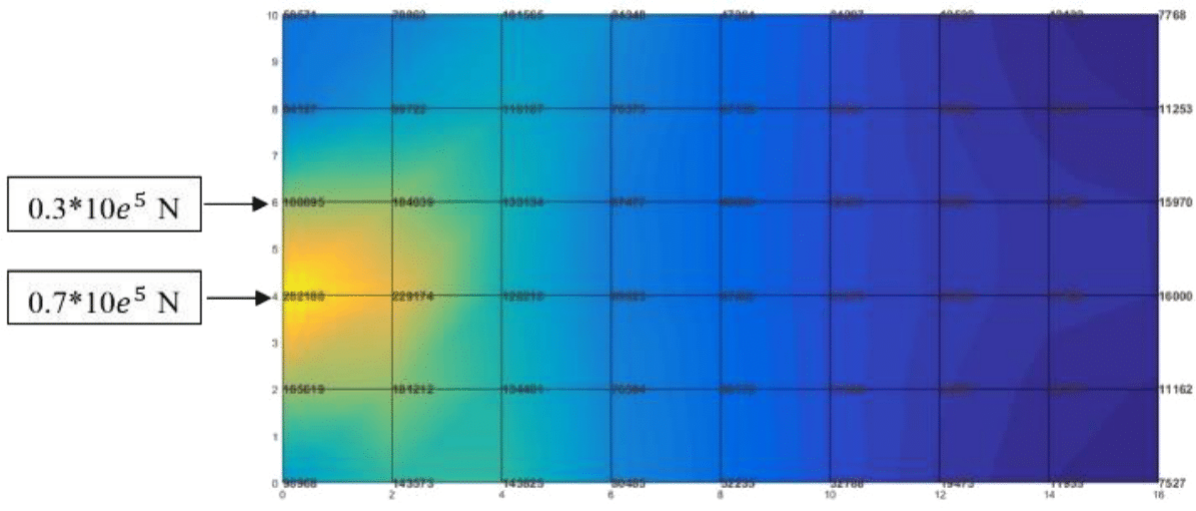
The results of the FEM stress analysis of the example 1, obtained in the finite element software environment.

Recalling the contents presented in the sections COI policy model, its numerical example and finite element method, to use the stress flux analogy for storage assignment problems, according the conceptual model, the area in example 1, considered as a plate with the same dimension and divisions, and the input/exit logs’ percentage of goods simulated as external forces in the same positions of the I/O points in the warehouse. The results show that, the stress distribution starts from the location with maximum stress concentration (load points), with maximum total potential energy towards the region which has the lowest stress with the minimum of potential energy (Fig. 9).

**Figure 10:**
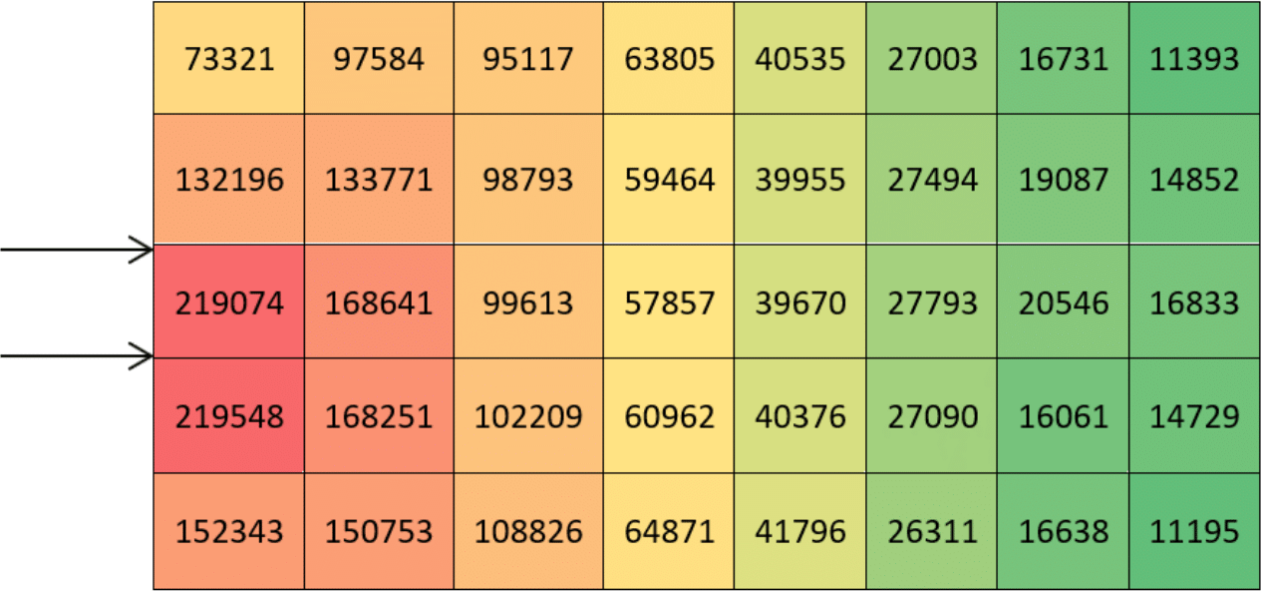
The results after calculating the mean stress of each element.

For more exact analysis, the average stress (calculated in the center of each element) shows that the elements with the maximum stress and highest potential energy are close to the loading points, and the lowest mean stress with lowest potential energy are exactly in counter (Fig. 10).

**Figure 11:**
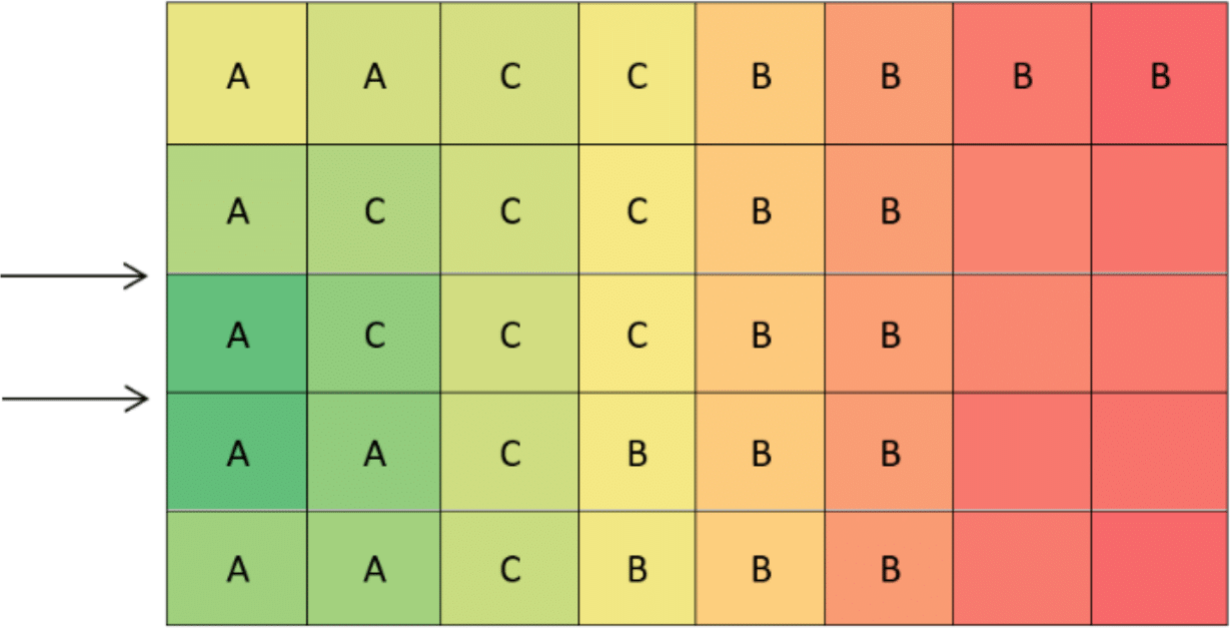
Storage assignment on after relocating according minimum stress (minimum potential energy).

**Figure 12:**
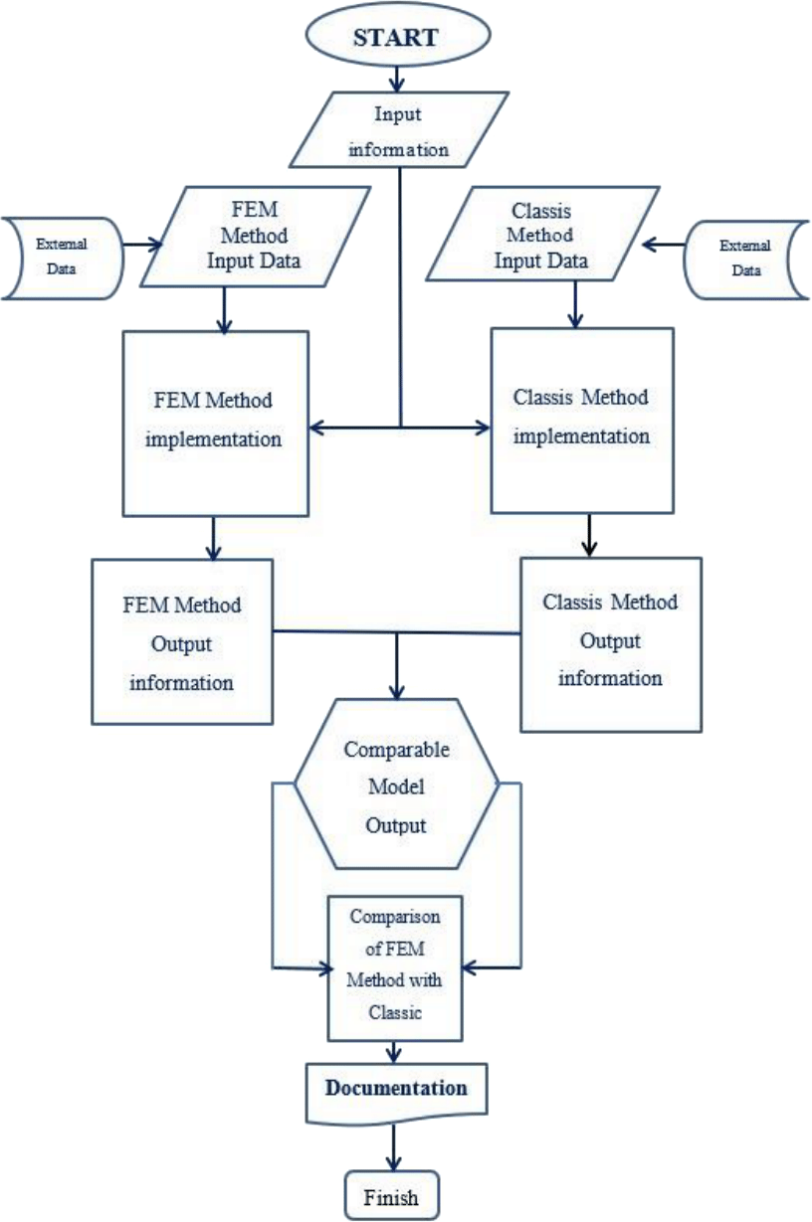
Schematic diagram of SAO/FEM algorithm implementation.

**Figure 13:**
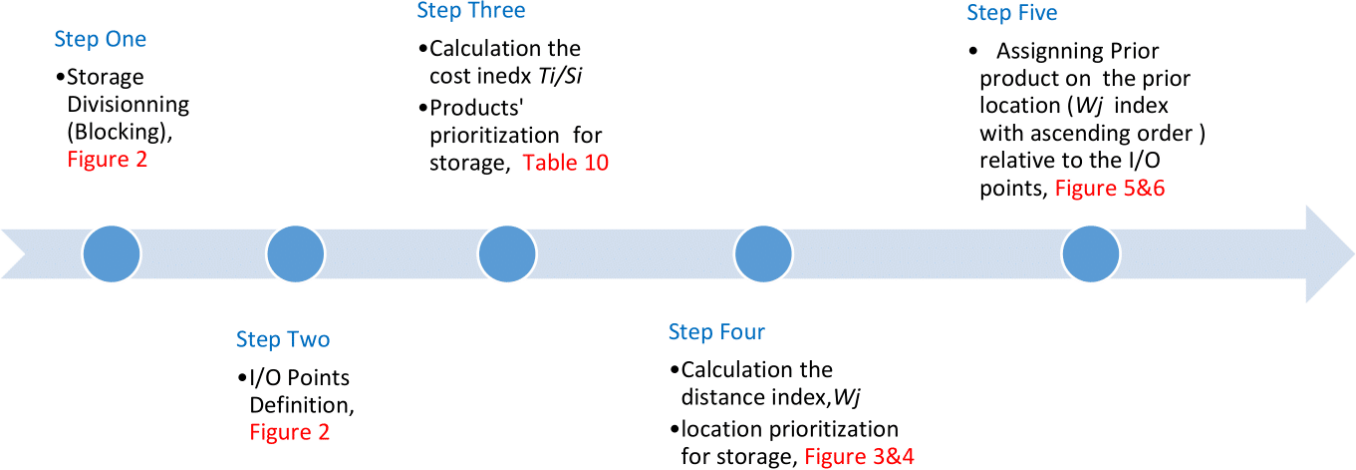
Classical model storage assignment procedure.

**Figure 14:**
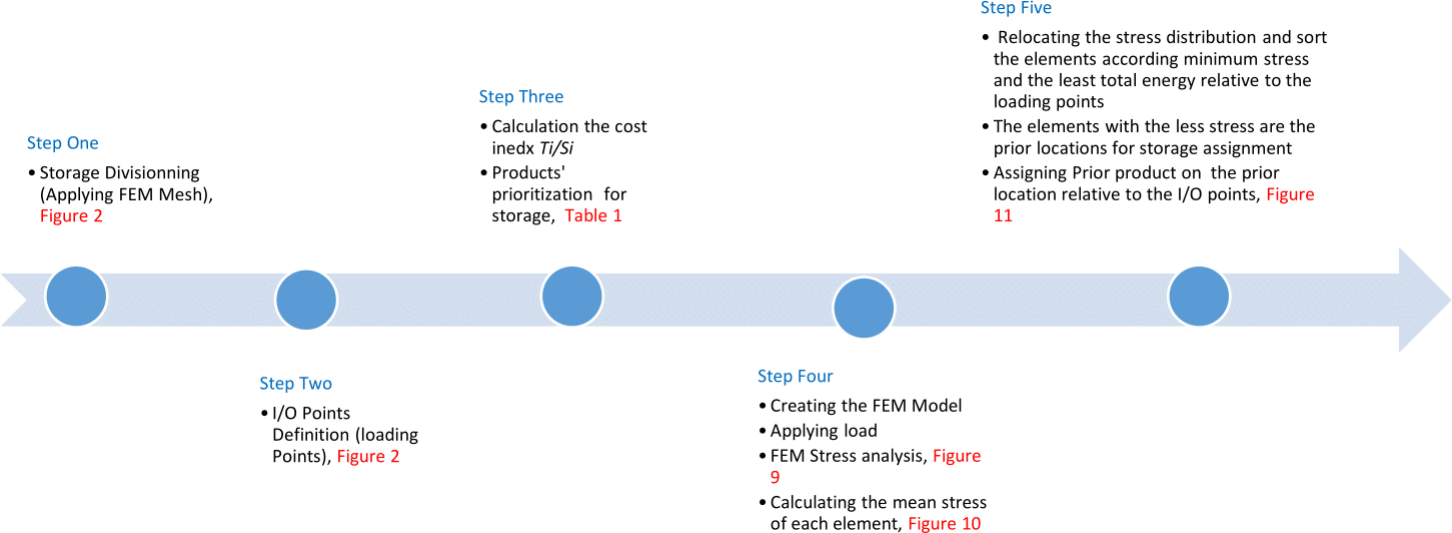
New model storage assignment procedure flow.

Comparing the result of the classical model (presented in Figs. 3 and 4 (‘Storage Assignment Example’)) with the new model (Fig. 10), clarify if the stress distribution calculations by finite element method relocate related to the I/O points, based on minimum stress and the least total energy, locations that have the preferences for storage assignment follow the same pattern as classical model and useable as a new model for storage optimization (Figs. 11 and 12). storage procedure diagrams in Figs. 13 and 14 show that both models have almost the same strategy up to step three but the procedure in step four and five are different.

### SAO/FEM Algorithm - application

The SAO/FEM algorithm (Fig. 12) designed to check the similarity of the two analogies to prove that storage assignment with the new algorithm is possible and have more advantages. SAO/FEM application is designed for solving the same problem in both algorithms simultaneously, analysis the output data, compare the results in a specific computer and show the capabilities of the new proposed algorithm and its advantages over the classical model.

**Figure 15:**
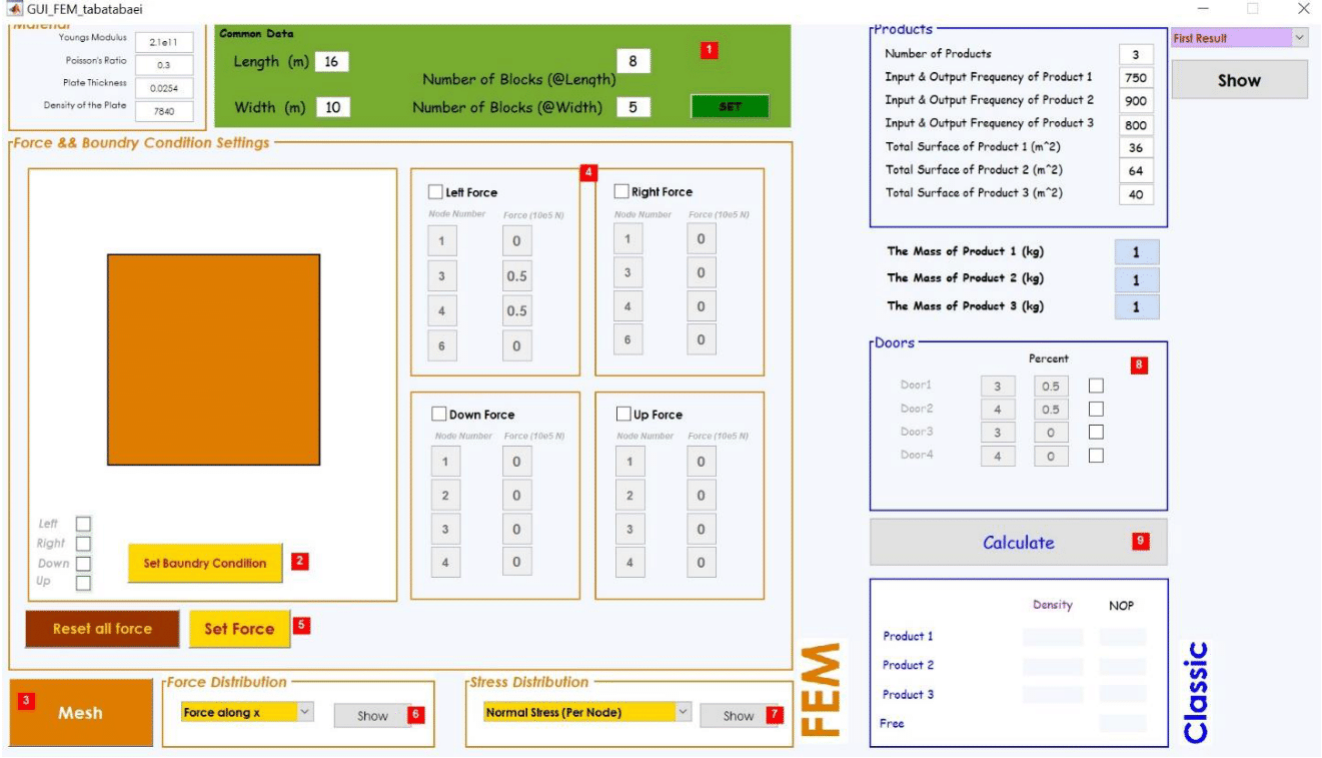
Graphical user interface of SAO/FEM application.

The graphical interface (GUI) shown in Fig. 15 designed for SAO/FEM application. It has three sections (Modeling, FEM and Classic) for data entry where the specifications data must be entered in order. In modeling part there are two sections, the first is the plate’s physical specifications and the second is dimensional information of the model in addition to its divisions biased on the unit of the length. In FEM part, boundary conditions apply to the area of the model, dimensions in no I/O point sections bounded. Then loads at the points that are equal to the I/O points proportional to the percentage of input and output of the goods apply to the model. In classical part, place of I/O points and each points’ entry and exit percentage of the goods define.

After data entry, the analysis phase starts, and software output shows the loads expansion and stress analysis (Since the elements with the maximum stress and the highest potential energy are close to the loading points, the lowest mean stress with lowest potential energy is exactly in counter).

After stress analysis, the application prepares a comparable model (Fig. 12) and calculate the average stress in the center of each element and then arrange the elements symmetrically. So, the elements with the minimum average stress and minimum potential energy, finally sort near the I/O point (virtual loading points) and display its graphs. Besides the application runs the classical calculations on the ideal assignment of goods in the warehouse performed and display its graph.

Then application calculate the index *Ti/Si* (Determination of each goods’ priority of storage) in both algorithms and arranges the prior goods on the elements from the lowest average stress to the highest in FEM model and from nearest distance to farthest distance in classical model. The next phase is comparison phase (Fig. 12). In this phase, SAO/FEM starts normalizing the result matrix of both algorithms and create the new matrixes with the elements calculate from 0 to 1 (independent of dimensions) for both. The graphical format of the results sample is shown in the Figs. 13 and 14, parts C, D.

Finally, SAO/FEM application compares the normal matrixes to calculate the conformity percentage of the performance of the storage assignment in both algorithms and calculating time. As observed in the SAO/FEM schematic diagram, the output of both methods must pass through data normalization filter to be able to be comparable with one another.

#### Graphical user interface simulation

Simulation is basically a set of assumptions about the function of the system within the framework of mathematical and logical relations. In this application, MATLAB software is used for computer simulation. Since this software enables easy access to use of matrix, arithmetic and functions, also the use of different algorithms as well as easy communication with different language programming, so it is highly desirable to implement algorithms that have mathematical complexity. Thus, in the study of the software and its well user-friendly capabilities, it has been used to implement the finite element method and integration of the classic method.

Communicate in a graphic, highly efficient format, and this is an attempt to escape the text settings that have to run all instructions and actually enter and exit the data through the program text without treating a GUI.

#### Numerical examples

In order to verify the capabilities of the proposed solution, some numerical examples have been solved by SAO/FEM application and the results are compered. SAO/FEM application solves each problem simultaneously in both algorithms, calculates the similarity of the performance along with the solution times.

**Figure 16:**
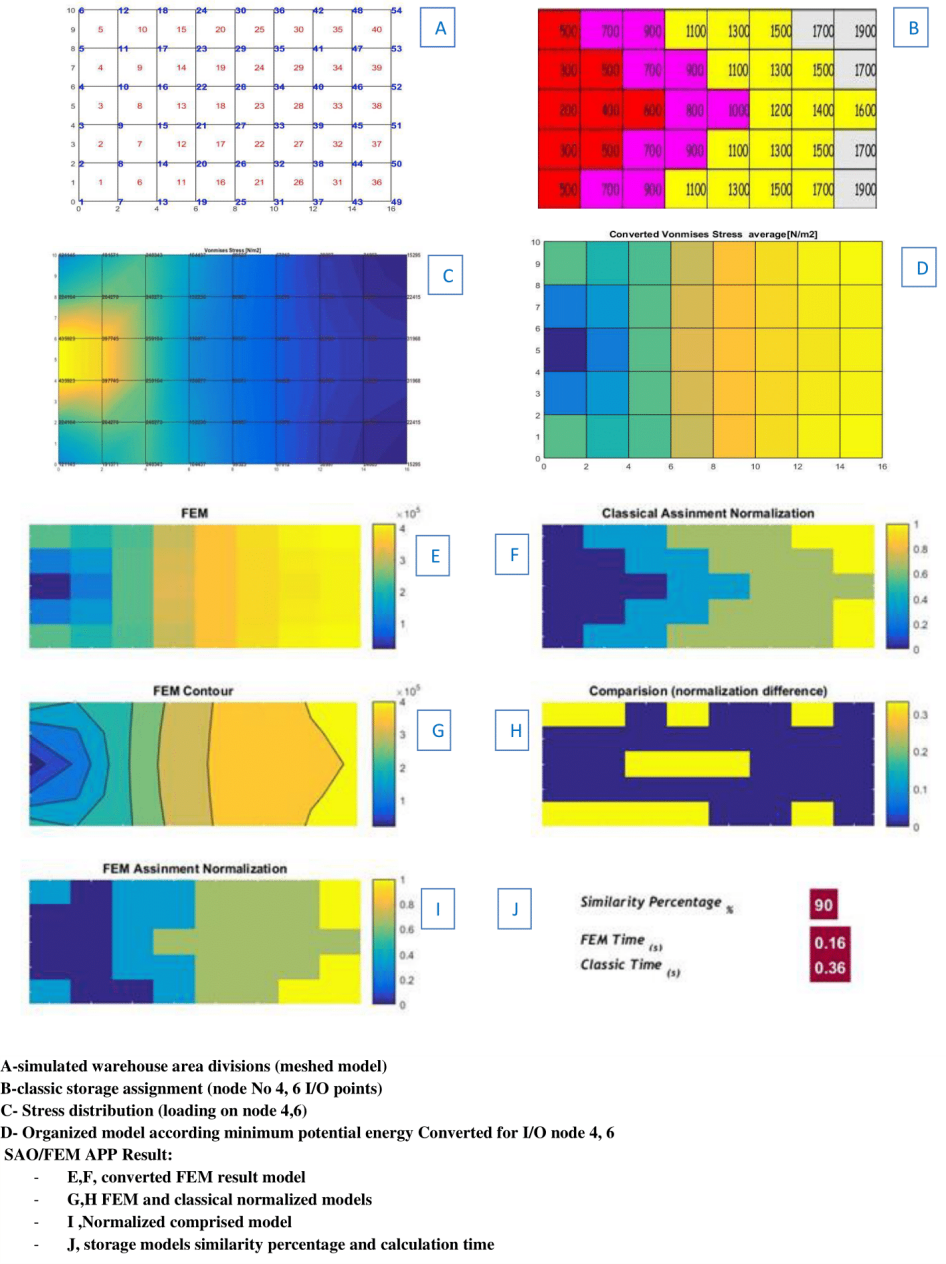
Scenario 1 solved manually separately by classic and proposed algorithms.

It is notable that the verification started with the scenario 1, in this scenario SAO/FEM has solved the same problems discussed in ‘Simulation and analysis’. Which have been solved manually by classic and separately by FEM software (the storage area of this problem has divided in to 40 similar elements). The SAO/FEM results shows that storage assignment performance of the FEM model is 90% similar to the classical model and has the better performance in calculation time (Fig. 16).

In scenario 2, SAO/FEM application solved the storage problem with the storage area 4 times larger than the scenario 1 (the storage area has divided in to 160 similar elements).

**Figure 17:**
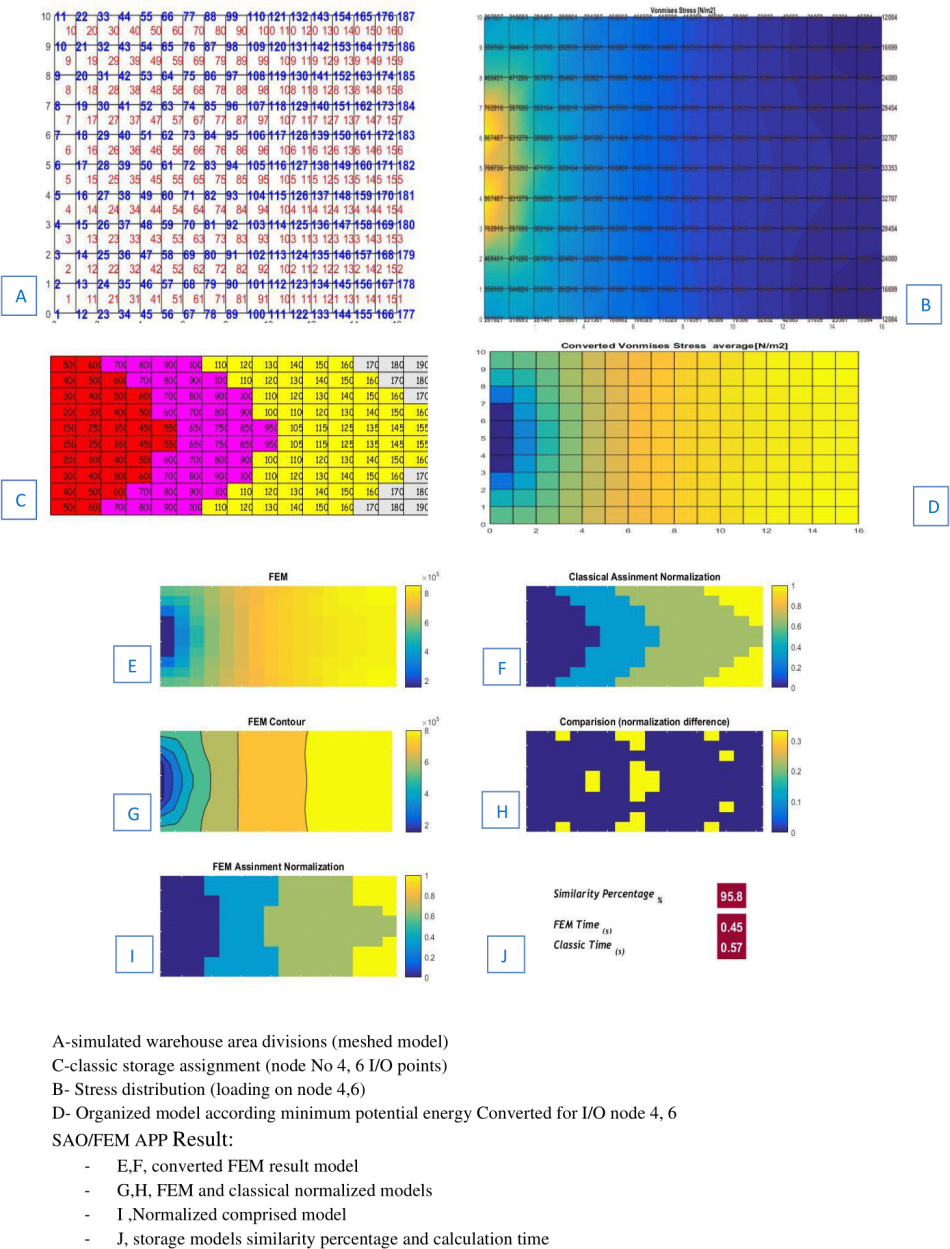
Scenario 2 solved by dividing the level of the warehouse into smaller divisions.

The SAO/FEM results shows that storage assignment performance of the FEM model is 95.8% similar to the classical model and has the better performance in calculation time (Fig. 17).

**Figure 18:**
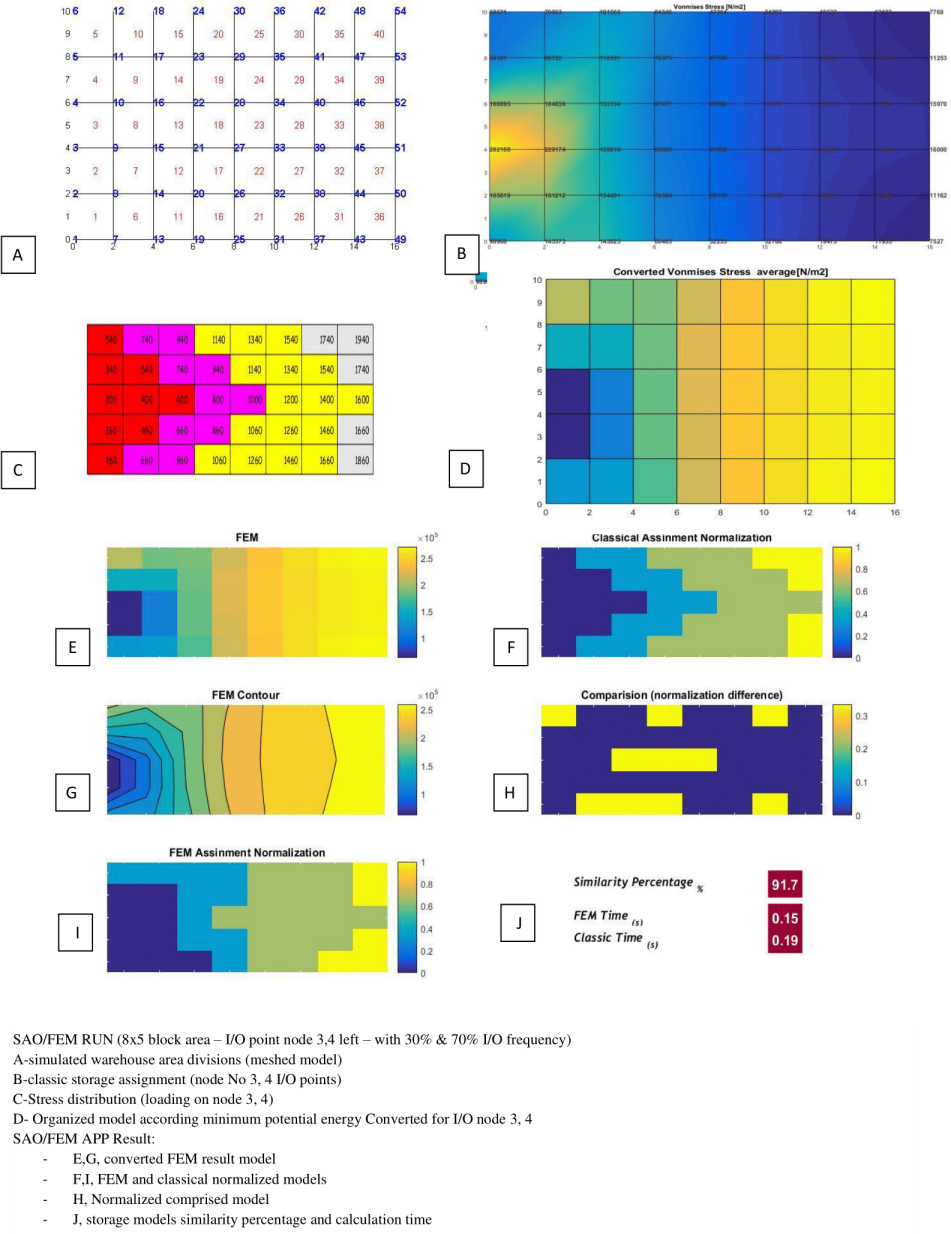
SAO/FEM results for scenario 1 in Table 4.

**Figure 19:**
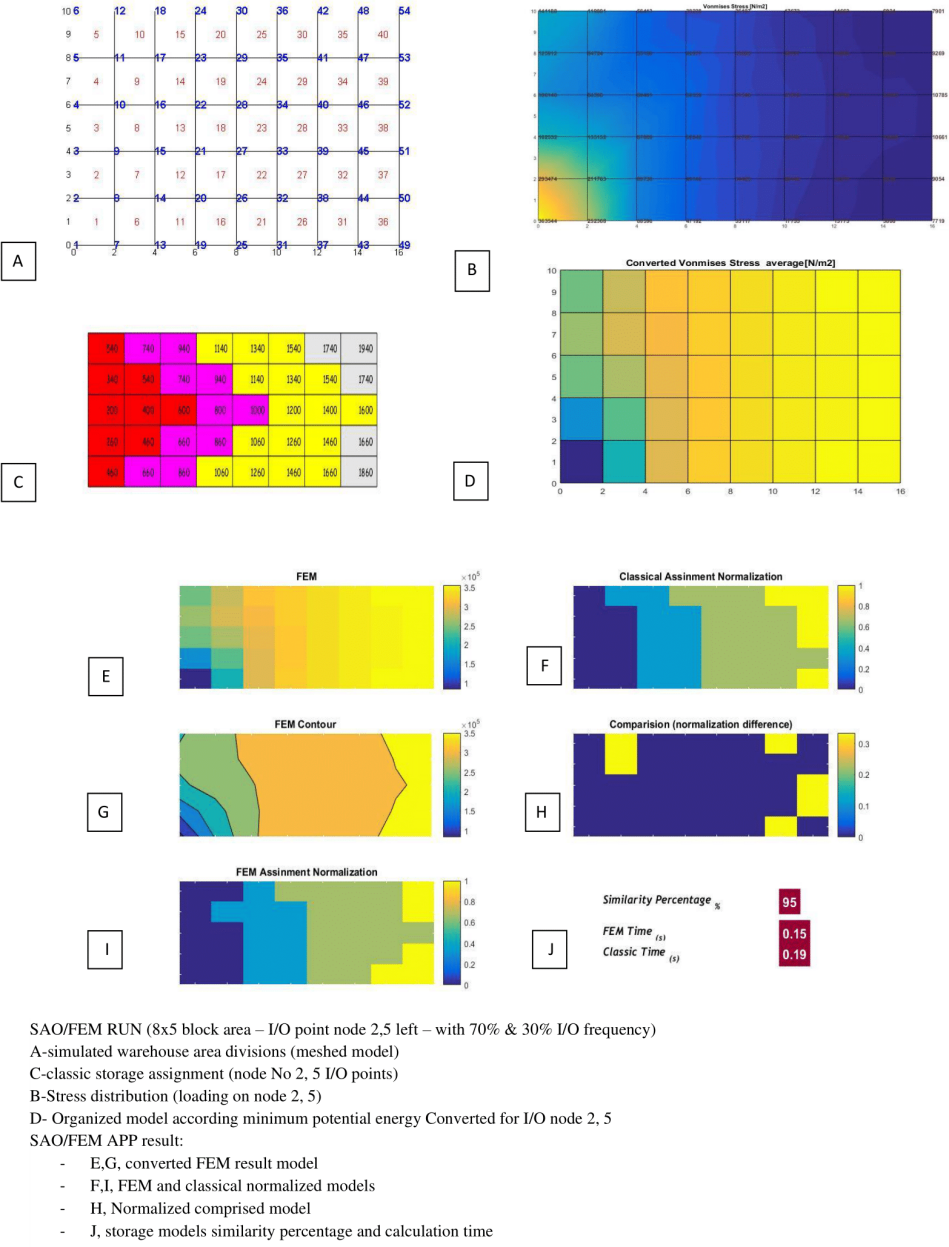
SAO/FEM results for scenario 3 in Table 4.

**Figure 20:**
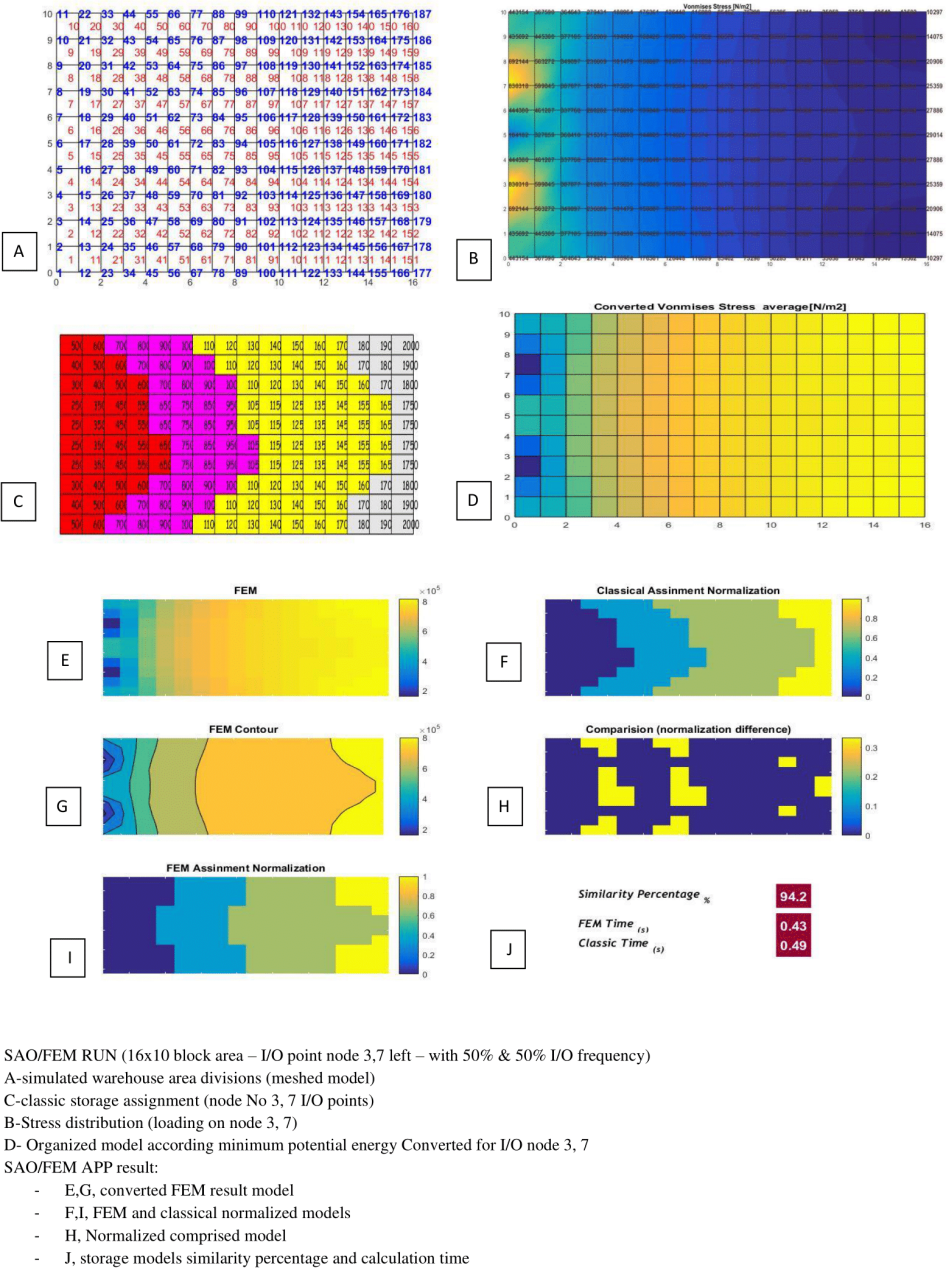
SAO/FEM results for scenario 4 in Table 4.

**Figure 21:**
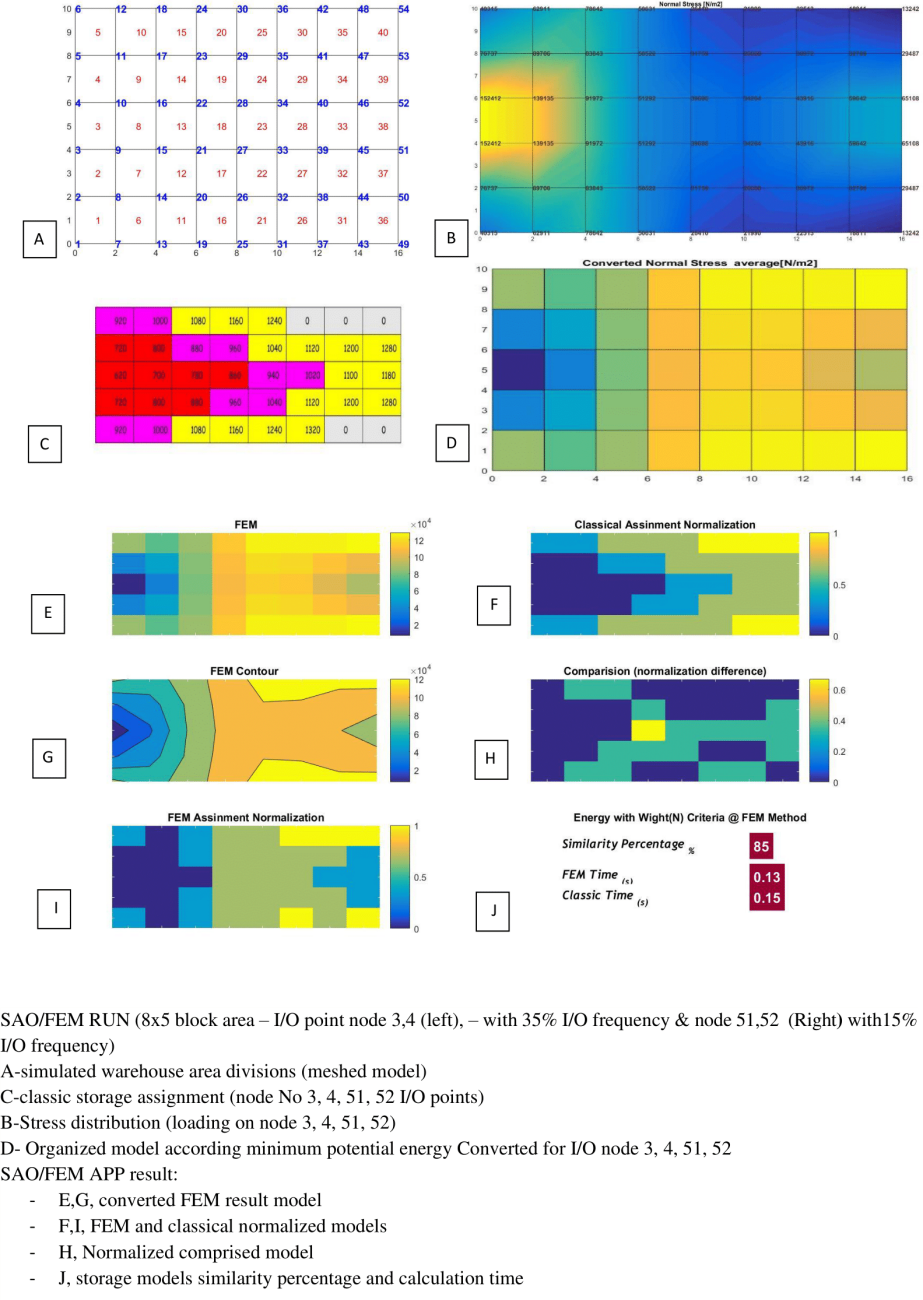
SAO/FEM results for scenario 1 in Table 5.

**Figure 22:**
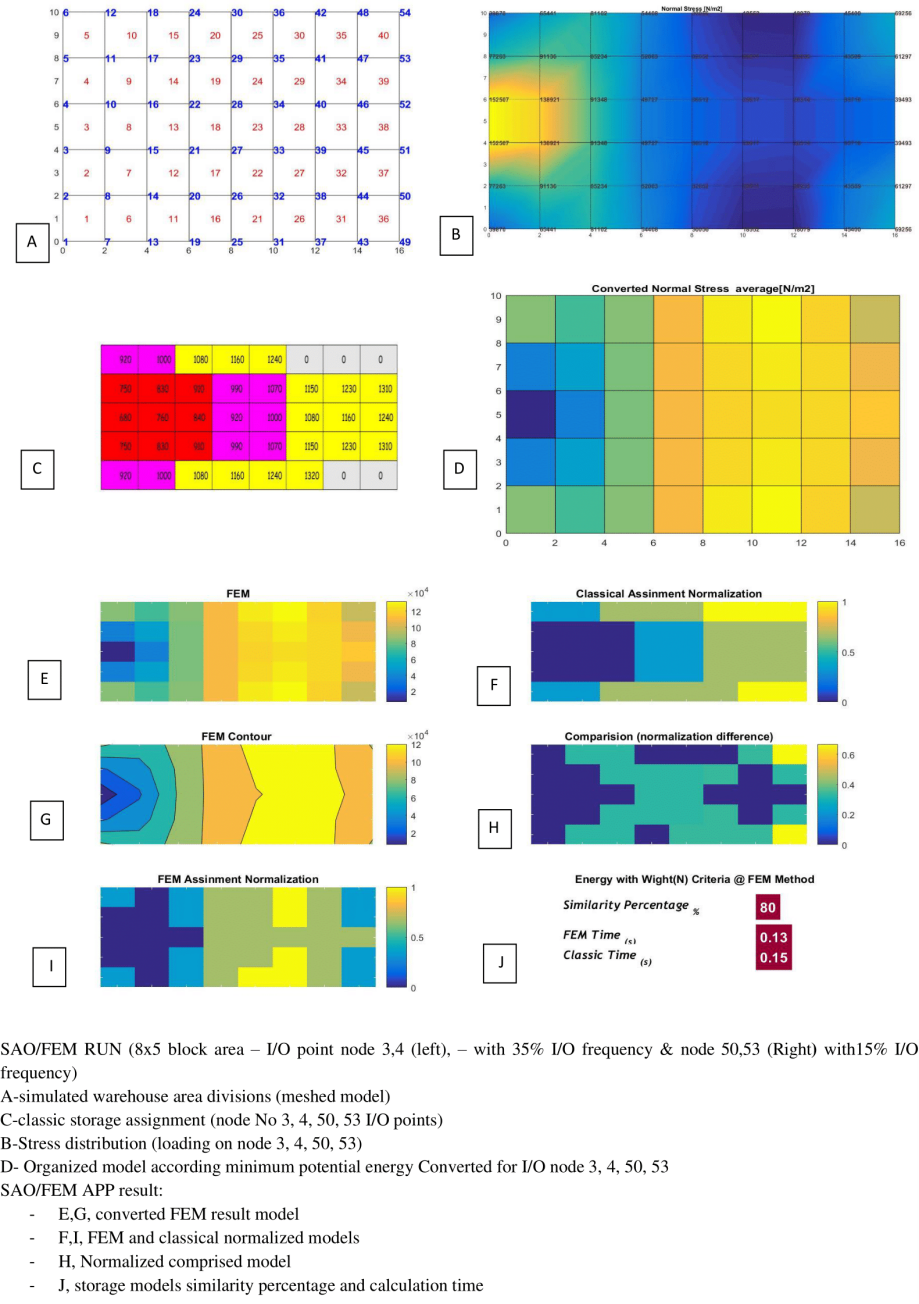
SAO/FEM results for scenario 2 in Table 5.

Tables 3 and 4 show the SAO/FEM application’s different outputs of different problems and confirm the better performance of the FEM model. Figs. 18–22 demonstrate the storage assignment performance of the proposed FEM model and its similarity results to the classical model for scenarios in Tables 4 and 5. The results show that SAO/FEM method is successful for storage assignment optimization, and has more capabilities and advantages:

1. Higher computational speed
2. More computational accuracy
3. The ability to prioritize the arrangement without the restrictions of the classical model
4. Ability to apply any changes to storage priority in the shortest time

As a conclusion, in solving the storage assignment problems, the SAO / FEM method is efficient and more effective than the classic model.

**Table 3:** SAO/FEM applicationresult for storage assignment with different order (example 2&3).

No	Mesh generation	I/O position	I/O frequency	FEM & Classic storage assignment similarity percentage	Classic time	FEM time
1	5*8 = 40	Node 4,6 (left side)	Both 50%	90%	0.36 s	0.16 s
2	10*16 = 160	Node 4,6 (left side)	Both 50%	95.8%	0.57 s	0.45 s

**Table 4:** SAO/FEM application result for storage assignment with different order (index examples).

No	Mesh generation	I/O position	I/O frequency	FEM & Classic storage assignment similarity Percentage	Classic time	FEM time
1	5*8 = 40	Node 3,4 (left side)	30% 70%	91.7%	0.19 s	0.15 s
2	5*8 = 40	Node 2,5 (left side)	50% 50%	91.7%	0.19 s	0.15 s
3	5*8 = 40	Node 2,5 (left side)	30% 70%	95%	0.19 s	0.15 s
4	10*16 = 160	Node 3,7 (left side)	Both 50%	94.5%	0.49 s	0.43 s

## Conclusions

This paper has opened a new topic in solving the storage assignments problems by an application inspired from a powerful computing method such as finite element. The finite element first analyzes the problem with the logical assumptions then, after obtaining the differential equation, solve the problem by numerical methods. In the finite element method, the mass considers as continuum area and divides it to simpler geometric shapes. Define the shape functions that satisfy the boundary conditions the equations of the elements calculated and the limits and the results obtained.

The advantages of the finite element method are the easy to use, speed and look with the approach of its continuity to the issues that have shown interesting results in solving the problems, while it has high ability in discretization, partial solution and analyzes the problem. The classic model determines the ideal assignment with cost equation calculation, which is based only on storage distance from the I/O points and does not rank the distribution. However, the proposed model focuses on the distribution priority for the goods on the storage points, and it is capable to indefinitely number/type of goods (independent of the coefficients and frequency of consumption of goods) with the proper bordering of the warehouse, for the ideal storage priority arrangement. And this calculation is practically difficult for more than three types/products in the classic model, so it is difficult to solve the problem. Also, the product storage flexibility and mathematical tools developed in this direction, along with comparing the problem-solving time to the classical method in the specified order, will further clarify this method’s applicability. The main issue in the accuracy of the results of the finite element method is the size of the grid and the type of element chosen for solving. The type of elective element is not important for assignment of goods in the warehouse, but the size and boundaries of the used grid size (in mesh generation) is important due to the type of warehouse problem in which the divisions determined based on the dimensional specifications of the goods, and since there is not geometric complexity (the warehouse surface is considered flat at the level of storing), the method will have a high accuracy in calculations.

In this paper, a new method called SAO/FEM introduced for storage assignment optimization. And an application based on the new algorithm with respect to the finite element method and minimum potential theorem designed and implemented, and to prove its capabilities, compared to the classic model, with the common problems have been solved. Creating a graphical user interface (GUI interface) is also an effort to enable the computer user to communicate in a graphic, highly efficient format, and this is trying to escape the text settings that have to run all instructions and actually enter and exit the data through the program text without treating a GUI.

Future research studies can consider:

**Table 5:** SAO/FEM application result for storage assignment with 4 parallel I/O position with deffirent frequency and distances (index examples).

No	Mesh generation	I/O position	I/O frequency	FEM & Classic storage assignment similarity Percentage	Classic Time	FEM Time
1	5*8 = 40	Node 3,4 (left side) Node 51,52 (Right side)	35% 35% 15% 15%	85%	0.15 s	0.13 s
2	5*8 = 40	Node 3,4 (left side) Node 50,53 (Right side)	35% 35% 15% 15%	80%	0.15 s	0.13 s

1. SAO/FEM algorithm optimization for better value added in terms of sustainability (energy), storage adjusted for weight characteristics of the different inventories and traveling WORK done with respect to minimum total potential energy definitions.
2. Optimization of SAO/FEM algorithm by changing I/O points’ locations (opposite or adjacent) or I/O Points intermediate distances or grid/mesh size consideration.
3. Practically validation of SAO/FEM algorithm, by using the warehouse information of the companies at the level of the third or fourth generation of the Industry

Also, the flexibility of the product storage and mathematical tools developed in this direction (with the definition and application of uniform force), along with comparing the problem-solving time to the classical method in the specified order, will further clarify the applicability of the proposed method in this paper.

## Additional Information and Declarations

### Data Availability

The following information was supplied regarding data availability: Raw data is available at Figshare: Fatahi Valilai, Omid (2020): Data set used in case study. figshare. Dataset. https://doi.org/10.6084/m9.figshare.13299269.v1. Code is also available at Figshare: Fatahi Valilai, Omid (2020): inventory optimization code. figshare. Software. https://doi.org/10.6084/m9.figshare.13299278.
